# Pulmonary Toxicity and Biodistribution of Sodium Fluoride in Male Rats Following Intratracheal Instillation and Nose-Only Inhalation Exposure

**DOI:** 10.3390/toxics14080688

**Published:** 2026-08-03

**Authors:** Tanya Bhatt, Dong Hyun Kim, Soyeon Jeon, Se-Hwan Choi, Jaebaek Jang, Mihye Kwon, Jang Woo Park, Min-Seok Kim, Iljung Lee, Wan-Seob Cho

**Affiliations:** 1 Department of Health Sciences, The Graduate School, Dong-A University, 37, Nakdong-daero 550 beon-gil, Saha-gu, Busan 49315, Republic of Korea; 2 Korea Radioisotope Center for Pharmaceuticals (KRICP), Korea Institute of Radiological & Medical Science (KIRAMS), 75 Nowon-ro, Nowon-gu, Seoul 01812, Republic of Korea; 3 Inhalation Toxicity Research Group, Korea Institute of Toxicology, Jeongeup-si 56212, Republic of Korea

**Keywords:** fluoride, lung inflammation, biodistribution, hazard, radiotracer

## Abstract

Pulmonary exposure to sodium fluoride (NaF) may occur in certain consumer-product and occupational settings, but its pulmonary toxicity and systemic biodistribution remain insufficiently characterized. This study investigated acute pulmonary inflammatory responses and biodistribution kinetics in rats following a single airway exposure to NaF. Non-radioactive NaF was administered in 7-week-old male Sprague-Dawley rats by intratracheal instillation at 0.75, 1.5, or 3.0 mg/rat, and pulmonary responses were evaluated at 1, 7, and 28 days using bronchoalveolar lavage fluid analysis and histopathology. Biodistribution was assessed using positron emission tomography/computed tomography (PET/CT) and organ gamma counting after administration of fluorine-18 radiolabeled sodium fluoride ([^18^F]NaF). NaF induced acute pulmonary inflammation characterized by increased neutrophils and eosinophils, elevated lactate dehydrogenase activity, and increased levels of tumor necrosis factor-alpha and eotaxin. These inflammatory responses gradually resolved by 7 and 28 days after exposure. PET/CT analysis showed rapid pulmonary clearance of NaF, with concurrent accumulation in bone and predominant urinary excretion within approximately 30 min after administration. These findings indicate that single airway exposure to NaF induces acute but reversible pulmonary inflammation despite rapid lung clearance, and call for evaluation under repeated or exposure-relevant conditions and in both sexes, as pulmonary toxicity was assessed only in male rats and only by intratracheal instillation.

## 1. Introduction

Chemicals used in household products, which are extensively employed in residential, occupational, and communal settings, pose significant risks to human health and the environment through multiple routes of exposure. The fatalities associated with the use of humidifier disinfectants in Korea have heightened both public awareness and regulatory scrutiny of chemical safety in consumer settings [1,2]. As a result, the safety evaluation of household chemicals has drawn increasing attention, especially for inhalable formulations such as sprays and aerosols. These products have been prioritized in toxicity testing frameworks due to accumulating evidence that highlights severe adverse effects following inhalation exposure [2,3]. The global outbreak of coronavirus disease 2019 (COVID-19) has further accelerated the use of personal hygiene and disinfection products within households, resulting in increased exposure to aerosolized chemical agents [4]. Although these products are effective in controlling microbes, their indiscriminate use can disrupt the ecological balance and, paradoxically, pose risks to human health. These concerns underscore the need for comprehensive toxicological assessments of chemicals used in household products, especially those designed for airborne application.

Among the various chemical agents used domestically, fluoride is of particular interest due to its natural abundance and wide range of applications. Fluoride is the 13th most abundant element in the Earth’s crust, accounting for approximately 0.059% by weight [5]. It is utilized across multiple sectors, including medicine, semiconductor manufacturing, energy materials, optical telecommunications, and liquid crystal technologies. In the medical and public health domains, fluoride is recognized for its cariostatic effects and has been incorporated into community water fluoridation programs in several countries, notably the United States, as a preventive measure against dental caries [6]. However, the widespread application of fluoride has also sparked considerable scientific and public debate due to its associated health risks. Documented adverse effects include dental fluorosis, neurotoxicity, and skeletal malformations, particularly under chronic or excessive exposure conditions [7,8,9]. To mitigate these risks, the World Health Organization has established a guideline limit of 1.5 mg/L for fluoride concentration in drinking water to minimize health risks while preserving its dental benefits [10]. In addition to water supplies, fluoride compounds are commonly found in oral hygiene products, such as toothpaste and mouthwash, with sodium fluoride (NaF) being one of the most widely used agents in these applications [11].

While fluoride in oral care products is primarily intended for dental benefits, it can also cause systemic exposure, not only through ingestion but potentially via mucosal absorption and incidental inhalation during product use or in occupational settings [12,13]. In occupational settings, inhalation exposure to fluoride can occur during manufacturing processes involving fluoride-containing compounds, such as those used in the production of aluminum or semiconductors [12]. To limit such exposure, occupational exposure limits for fluorides (as F) have been established, including a US Occupational Safety and Health Administration (OSHA) permissible exposure limit and a National Institute for Occupational Safety and Health (NIOSH) recommended exposure limit of 2.5 mg/m^3^ (8-h time-weighted average), consistent with the American Conference of Governmental Industrial Hygienists threshold limit value of the same magnitude [14,15]. Despite these limits, historical workplace monitoring has documented airborne fluoride concentrations of 11–24 mg/m^3^ among chronically exposed workers, with higher concentrations regarded as elevated [16]. In consumer settings, inhalation exposure can occur through daily activities, such as using fluoride-containing oral care products (e.g., toothpaste and mouthwash) and showering or bathing with fluoridated water. These exposures may occur when fluoride-containing solutions are aerosolized, for example, as fine droplets generated during tooth brushing or mouth rinsing, or when fluoridated water is dispersed through showers, humidifiers, or spray devices. In addition, NaF and other soluble fluorides are incorporated into a range of consumer and industrial products, including certain cleaning agents, wood preservatives, and antimicrobial formulations, some of which may be applied as sprays or aerosols [16]. In contrast to occupational environments, however, airborne NaF concentrations arising from such consumer uses have rarely been quantified. Although inhalation exposure to fluoride is common across various settings, its potential to induce lung inflammation and its biodistribution remain poorly understood. Hence, to address this gap, this study evaluated pulmonary inflammation induced by NaF and related mechanisms using a rat intratracheal instillation model. Additionally, the biodistribution of NaF was evaluated using positron emission tomography/computed tomography (PET/CT) and gamma counting in organs of rats with [^18^F]NaF as a radiotracer.

## 2. Materials and Methods

### 2.1. Preparation of [^18^F]NaF Radiotracer

Radiochemical synthesis of [^18^F]fluoride ([^18^F]F^−^), a crucial radionuclide for PET imaging, was performed via the ^18^O(p, n)^18^F nuclear reaction. This process involved irradiating a ^18^O-enriched water target with a proton beam. The irradiation was conducted using a PETtrace 16.5 MeV cyclotron from GE Healthcare (Uppsala, Sweden), a medical cyclotron designed to produce radioisotopes. A proton beam current of 30 µA was applied for 15–20 min, yielding 18.5–22.2 GBq of radioactivity, which provided a sufficient quantity of [^18^F]F^−^ for subsequent radiopharmaceutical synthesis, satisfying both clinical and research requirements. Following its generation, the [^18^F]F^−^ dissolved in the enriched ^18^O-water was rapidly transferred under nitrogen gas pressure to a radiation-shielded TRACERlab FX2 N automatic synthesis module (GE Healthcare, Chicago, IL, USA). This automated system minimized occupational radiation exposure and ensured process reproducibility and adherence to good manufacturing practice (GMP). Within the module, the [^18^F]F^−^ underwent a purification step. It was retained by a Sep-Pak light Quaternary Methyl Ammonium (QMA) anion-exchange cartridge, which selectively bound the fluoride anion, thereby separating it from non-radioactive constituents and the bulk aqueous phase. Subsequently, the purified [^18^F]F^−^ was eluted from the cartridge with 6 mL of 0.9% NaCl saline solution, yielding the final [^18^F]NaF product. The radiochemical purity and radioactivity of [^18^F]NaF in saline were assessed by radio-thin-layer chromatography (radio-TLC) using 95:5 (*v*/*v*) acetonitrile:water as the mobile phase and by a CRC-55tPET dose calibrator (Capintec, Ramsey, NJ, USA), respectively.

### 2.2. Animal Condition and Care

The toxicity studies were conducted at Dong-A University, while the biodistribution studies were performed at the animal research facility of the Korea Institute of Radiological and Medical Sciences (KIRAMS). All animal experiments were conducted in accordance with institutional and national guidelines for animal welfare and were approved by the respective Institutional Animal Care and Use Committees (IACUCs) (KIRAMS: KIRAMS 2022-0100 (approval date: 7 September 2022); Dong-A University: DIACUC-25-17 (approval date: 23 June 2025). Animals were maintained under standardized environmental conditions (temperature: 23 ± 1 °C; relative humidity: 50 ± 10%; 12-h light/dark cycle) and were allowed a 1-week acclimation period prior to experimental procedures. Food and water were provided *ad libitum* throughout the study period. Animals were randomly assigned to experimental groups, and treatments and measurements were performed using the same procedures across groups. Potential confounders such as cage location were not specifically controlled beyond standard housing under identical environmental conditions. Group allocation was known to the personnel involved in animal allocation and treatment administration, as different groups required different dosing or exposure procedures. Outcome assessments and data analyses were performed according to predefined procedures. Complete blinding was not applied at all stages of the experiment.

### 2.3. Intratracheal Instillation of NaF in Rats to Assess Lung Inflammation

The pulmonary toxicity of NaF was evaluated in male Sprague-Dawley (SD) rats through bronchoalveolar lavage fluid (BALF) analysis and histological analysis at days 1, 7, and 28 after a single intratracheal instillation. The intratracheal instillation was performed according to our previously described method [17]. Briefly, six-week-old male SD rats, weighing 212 ± 9 g, were obtained from Central Laboratory Animal (Seoul, Republic of Korea) and acclimatized for one week. Rats were anesthetized using isoflurane (Piramal Critical Care, Bethlehem, PA, USA) via a rodent anesthesia system (VetEquip, Pleasanton, CA, USA). Then, NaF suspensions dispersed in distilled water (DW) were administered to the lungs of rats with intratracheal intubation using a 16-gauge polycarbonate catheter (BD Biosciences, San Jose, CA, USA). After a 7-day acclimatization period, rats weighing 223 ± 15 g were used for the acute lung inflammation study. Acute lung inflammation was evaluated 1 day after intratracheal instillation of NaF at doses of 0.75, 1.5, and 3.0 mg/rat (approximately 3.4, 6.7, and 13.5 mg/kg bw, respectively) in a total volume of 500 μL. The vehicle control group received the same volume of DW. Then, the chronic effects following a single exposure to NaF at 3.0 mg/rat were evaluated on days 7 and 28 post-exposure to assess the reversibility of toxic effects.

### 2.4. Bronchoalveolar Lavage Fluid Analysis

At days 1, 7, and 28 after a single intratracheal instillation, the lungs were lavaged 4 times with ice-cold phosphate-buffered saline (PBS), and the collected BALF was centrifuged at 250× *g* for 5 min to separate the cellular components. The cells from 4 lavages were pooled by resuspending them in 1 mL of PBS containing 10% fetal bovine serum, and the total cell count was determined using a NucleoCounter (Chemometec, Allerod, Denmark). Then, cytospin slides were prepared at 4 × 10^4^ cells/slide. The cells were subsequently fixed with methanol for 5 min and stained with Diff-Quik (Thermo Fisher Scientific, Waltham, MA, USA). Then, the differential cell counting was performed by counting 300 cells per slide using a light microscope (Nikon, Tokyo, Japan).

### 2.5. Measurement of Biochemical Parameters and Pro-Inflammatory Cytokines in BALF

Biochemical and cytokine analyses were conducted using the supernatant obtained from the first BALF. Lactate dehydrogenase (LDH) activity was quantified using the Cytotoxicity Detection Kit (Roche Diagnostics, Mannheim, Germany) according to the manufacturer’s instructions, and results were expressed as fold change relative to the vehicle control. The total protein concentration in BALF was determined using a bicinchoninic acid (BCA) assay (Thermo Fisher Scientific) following the manufacturer’s protocol. The levels of pro-inflammatory cytokines, including interleukin (IL)-1β, IL-6, tumor necrosis factor (TNF)-α, eotaxin, and cytokine-induced neutrophil chemoattractant-3 (CINC-3), were measured using commercial enzyme-linked immunosorbent assay (ELISA) kits according to the manufacturer’s instructions (DuoSet^®^; R&D Systems, Minneapolis, MN, USA).

### 2.6. Histological Analysis

For histological evaluation, lungs were fixed in 10% neutral-buffered formalin and processed for paraffin embedding according to standard histological procedures. The slides were stained with hematoxylin and eosin and subjected to histopathological analysis under a light microscope (Nikon).

### 2.7. In Vivo Distribution of [^18^F]NaF in Rats Following Two Different Respiratory Exposures

Nose-only inhalation and intratracheal instillation were performed to evaluate the biokinetics of [^18^F]NaF in rats following pulmonary administration. The nose-only inhalation study was selected as a realistic inhalation exposure model, while intratracheal instillation was used to quantify lung deposition and biodistribution relative to the initial deposition level. This dual approach was intended to provide complementary information on the pulmonary biodistribution of NaF under realistic inhalation and controlled instillation conditions, rather than to establish a continuous dose-response relationship between the tracer imaging and toxicological studies.

#### 2.7.1. Inhalation Exposure of [^18^F]NaF in Rats

Six-week-old male SD rats (initial body weight: 215 ± 5 g) were acclimatized for 1 week, and at the time of exposure, their body weight was 229 ± 11 g. The animals were exposed to [^18^F]NaF aerosols using a nose-only inhalation system (VT-HOOF-12, VITALS, Daejeon Republic of Korea). Aerosolized [^18^F]NaF was generated via an impinging jet nebulizer, and aerosol concentration within the exposure chamber was monitored during the 60 min exposure period using a 1.0 μm Grade A-E binderless glass microfiber filter (LabExact, Hawthorne, NJ, USA). Particle size distribution was measured using a cascade impactor (Model 135-8B Mini-MOUDI, TSI, Shoreview, MN, USA), equipped with membrane filters corresponding to aerodynamic diameters of 0.18, 0.32, 0.56, 1.0, 1.8, 3.2, 5.6, and 10 μm, with aluminum foil substrates installed at each stage. The radioactivity captured on each filter was measured to determine the aerosol particle size profile. For aerosol generation, a radiolabeled [^18^F]NaF solution (37 MBq/mL) was nebulized at an airflow rate of 8 L/min and mixed with filtered air at 10 L/min, resulting in a total flow rate of 18 L/min. This total flow was distributed across the exposure ports at approximately 1 L/min per port for the 18 rats exposed simultaneously, providing a continuous flow-past supply of test aerosol at each nose port. The specifications of this type of nose-only exposure chamber have been described previously [18]. The mean aerosol concentration in the exposure chamber during the exposure period was 48.1 MBq/m^3^. The total amount of [^18^F]NaF aerosols administered to each rat was 0.74–1.11 MBq.

#### 2.7.2. Intratracheal Instillation of [^18^F]NaF in Rats

For intratracheal instillation, rats were anesthetized with isoflurane (Piramal Critical Care) using a rodent anesthesia system (VetEquip). Controlled pulmonary delivery was performed using a precision instillation device (Visual Instillobot, Sejongbio, Daejeon, Republic of Korea), with each animal receiving 3.7 MBq of [^18^F]NaF in 0.5 mL of saline. At 5, 30, 60, 90, and 120 min after administration, animals were euthanized via CO_2_ inhalation, and biological samples, including blood, heart, lungs, liver, kidneys, spleen, stomach, muscle, small intestine, large intestine, brain, and bone, were collected and weighed. The radioactivity in each sample was measured using a 2480 WIZARD2 Automatic Gamma Counter (PerkinElmer Life and Analytical Sciences, Waltham, MA, USA), and results were expressed as the percentage of injected dose per gram of tissue (%ID/g).

### 2.8. PET/CT Imaging of [^18^F]NaF After Inhalation or Intratracheal Instillation in Rats

PET/CT imaging was performed using the NanoScan PET/CT system (Mediso Medical Imaging Systems, Budapest, Hungary) at the Korea Radioisotope Center for Pharmaceuticals (Seoul, Republic of Korea). The treatment doses for intratracheal instillation and inhalation were approximately 10 times higher than those for the automated gamma counter (i.e., instillation: 29.6–37.0 MBq/rat; inhalation: 7.40–11.1 MBq/rat). These administered radioactivities were selected to provide sufficient PET imaging signal and should not be interpreted as toxicologically equivalent to the chemical dose of the non-radioactive NaF used in the lung inflammation study. Dynamic PET scans were acquired for 90 min following the final administration of [^18^F]NaF, either after a 1 h nose-only inhalation exposure or immediately after intratracheal instillation. For the nose-only inhalation experiments, PET/CT acquisition and tissue sampling were initiated only after completion of the 1-h exposure period; therefore, these data represent post-exposure biodistribution and do not capture the earliest absorption phase during inhalation. CT images were acquired after the PET scan using an X-ray voltage of 50 kVp and a reference tube current-time product of 0.16 mAs. The total scan duration was approximately 6 min. The CT data were used for both anatomical localization and attenuation and scatter correction of the PET image. PET data were reconstructed using the 3D ordered subset expectation maximization (3D-OSEM) algorithm. The dynamic PET data were divided into frames: 15 frames of 40 s each, 4 frames of 10 min each, and 2 frames of 20 min each, enabling detailed analysis of time-dependent changes. Attenuation correction was applied using the corresponding CT images. The reconstructed PET data analysis was conducted using the PET Model Organism Database (PMOD) software (version 3.8; PMOD Technologies Ltd., Zurich, Switzerland). Standardized uptake values (SUVs) were calculated based on each rat’s body weight, the administered radioactivity of [^18^F]NaF, and the defined volumes of interest (VOIs) for key organs, including the brain, lungs, liver, stomach, kidneys, intestines, and bone, to assess the biodistribution over time.

### 2.9. Statistical Analysis

Statistical analysis and graph preparation were conducted using GraphPad Prism software (version 10.5.0; La Jolla, CA, USA). Results are represented as mean ± standard deviation. To compare the two groups, an unpaired nonparametric Mann-Whitney U test was utilized. A one-way analysis of variance (ANOVA) followed by post-hoc Tukey’s tests was employed for multiple group comparisons. A *p*-value of less than 0.05 was considered statistically significant.

## 3. Results

### 3.1. Characteristics of Prepared [^18^F]NaF

**Figure 1:**
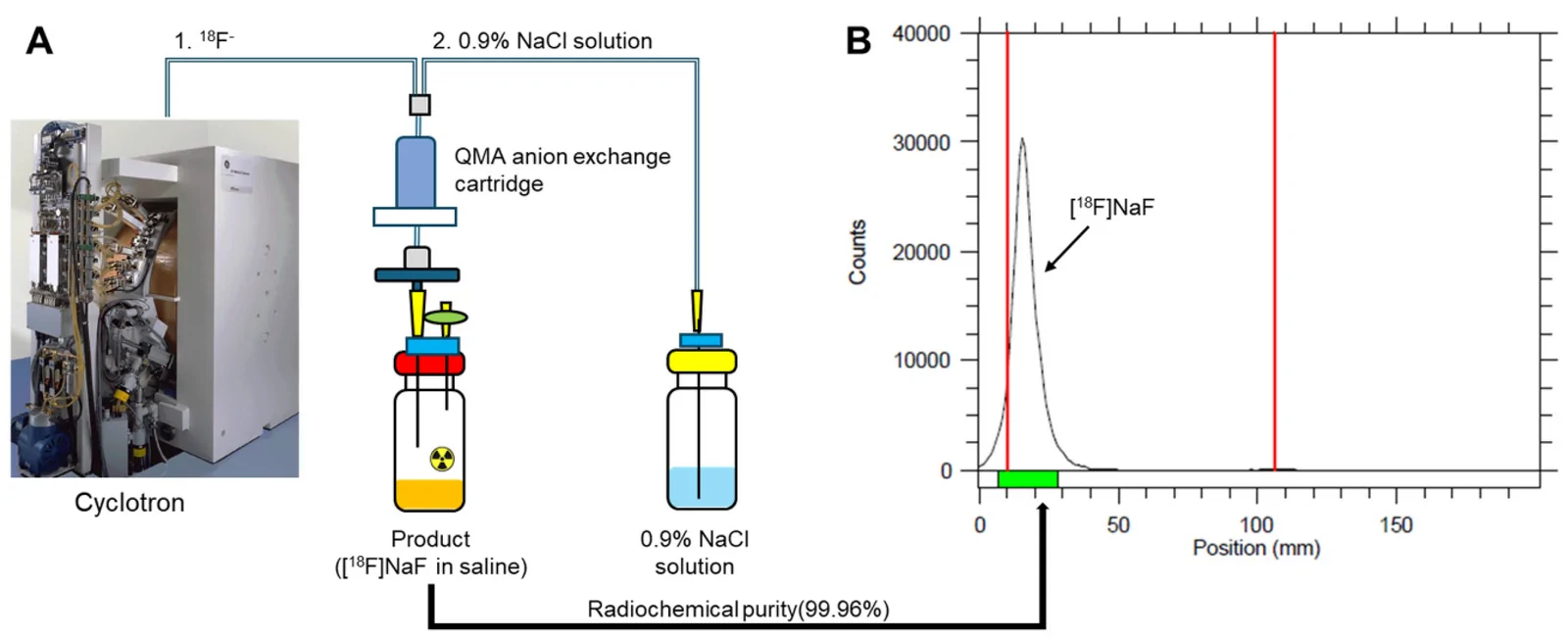
Characteristics of prepared [^18^F]NaF. (**A**) Schematic of [^18^F]NaF synthesis using an automatic synthetic module. (**B**) The radiochemical purity of [^18^F]NaF, evaluated by radio-TLC. The radiochemical purity of [^18^F]NaF was determined to be 99.96%. The vertical red lines indicate the origin (**left**, application point) and the solvent front (**right**) on the TLC plate. The green area highlights the integrated region used to quantify the radioactive peak of [^18^F]NaF.

An automated synthetic module was used for the large-scale, in-house production of [^18^F]NaF for this study, as illustrated in Figure 1A. The radiochemical yield ranged from 85% to 95%, and the radiochemical purity was consistently ≥99%, as confirmed by radio-TLC (Figure 1B). These values demonstrated that the synthesized [^18^F]NaF was of high quality and suitable for in vivo PET imaging studies.

### 3.2. Acute Lung Inflammation 1 Day After a Single Intratracheal Instillation of NaF

**Figure 2:**
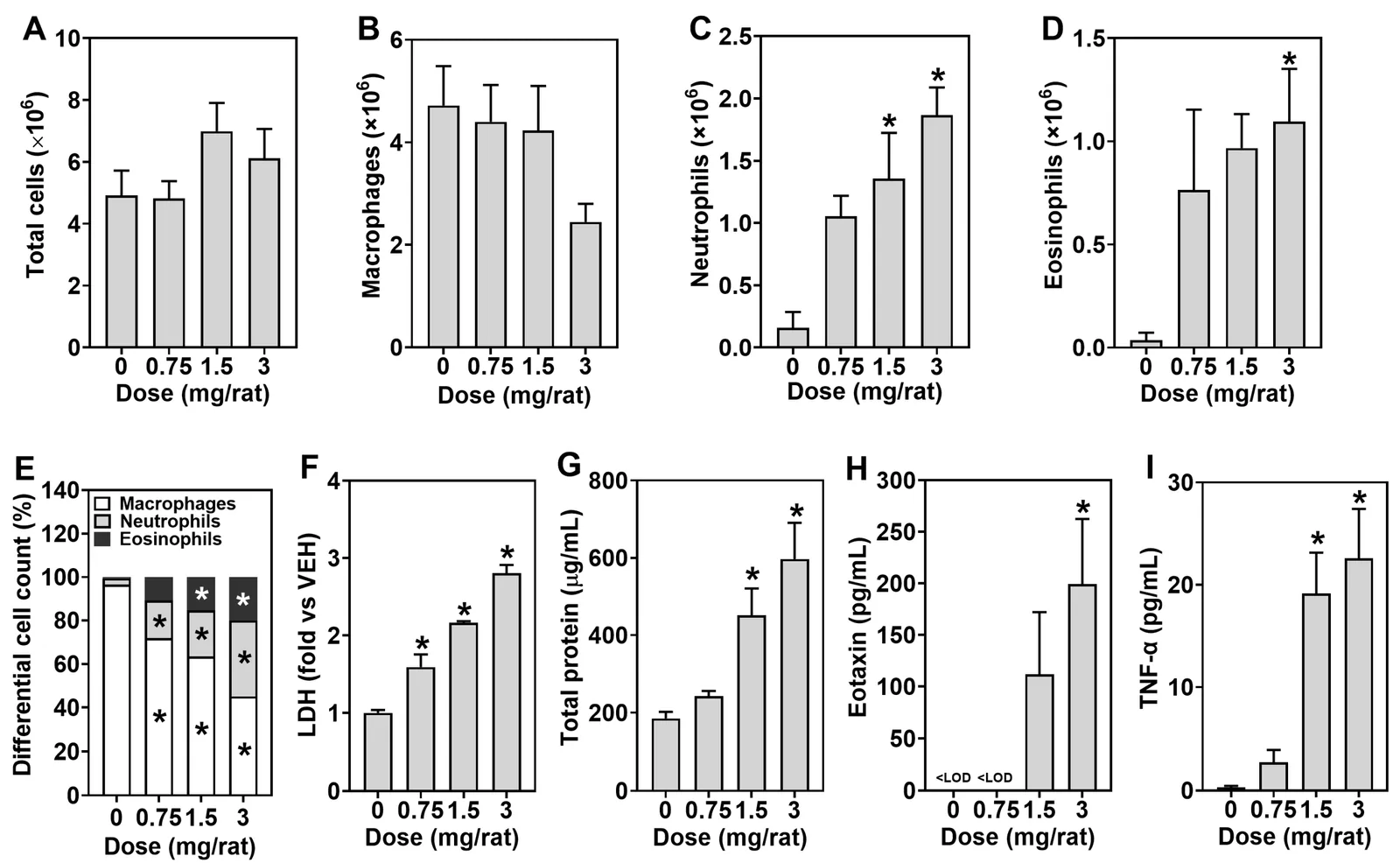
The levels of acute lung inflammation at 1 day after a single intratracheal instillation of NaF. NaF was administered at 0.75, 1.5, and 3.0 mg/rat, and lung inflammation was evaluated by the bronchoalveolar lavage fluid analysis. (**A**–**D**) The cytological analysis, including (**A**) the number of total cells, (**B**) the number of alveolar macrophages, (**C**) the number of neutrophils, (**D**) the number of eosinophils, and (**E**) the percentage of differential cell counts. (**F**,**G**) The biochemical analysis, including (**F**) the levels of lactate dehydrogenase (LDH) and (**G**) the levels of total protein. (**H**) The levels of eotaxin. In panel (**H**), <LOD indicates values below the limit of detection of the ELISA kit (7.81 pg/mL). (**I**) The level of TNF-α. The 0 mg/rat group corresponds to the vehicle control, which was administered 500 μL of distilled water. Data are mean ± standard deviation (*n* = 4 per group). * *p* < 0.05 compared to the vehicle control group.

**Figure 3:**
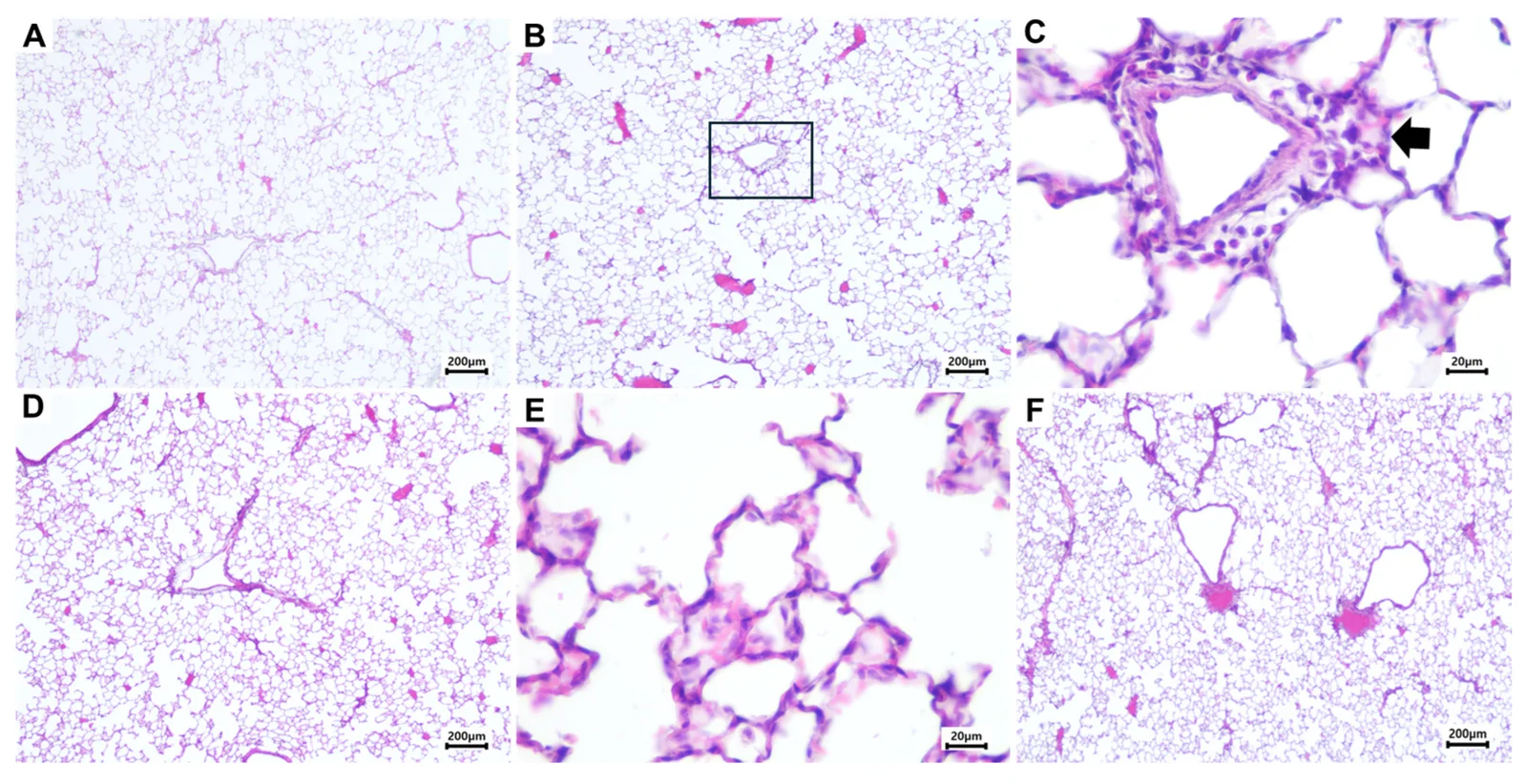
The histological evaluation of lung tissue after the intratracheal instillation of NaF in rats. Representative images of lung lesions at days 1, 7, and 28 after a single intratracheal instillation of NaF (3 mg/rat) in rats. (**A**) The lung lesion of the vehicle control at 1 day after instillation. Both (**B**) low-magnification (×40) and (**C**) high-magnification (×400) images of lung lesions 1 day after instillation of NaF at 3 mg/rat showed mild inflammatory cell infiltration in the alveoli and perivascular regions (arrow). The boxed area in (**B**) indicates the region shown at higher magnification in (**C**). However, (**D**) the low-magnification (×40) and (**E**) high-magnification (×400) views of lung lesions 7 days after NaF instillation at 3 mg/rat showed no histological alterations. (**F**) Lung lesion at 28 days after instillation of NaF at 3 mg/rat showed no tissue damage.

Acute lung injury in rats was evaluated by BALF analysis 1 day after intratracheal instillation. In BALF, total cell and alveolar macrophage counts did not differ significantly between any treatment group and the vehicle control group (Figure 2A,B). However, neutrophil counts were significantly higher in rats administered NaF at 1.5 and 3.0 mg/rat than in the vehicle control group (Figure 2C). Likewise, eosinophil counts were significantly increased in the high-dose group (3.0 mg/rat) compared with the vehicle control group, whereas no significant changes were observed at the lower doses (Figure 2D). Numerical differential cell counts were consistent with the percentage data, indicating a dose-dependent increase in neutrophil and eosinophil proportions, which together comprised approximately 60% of total BALF cells at 3.0 mg/rat (Figure 2E). The levels of LDH and total protein in BALF also increased in a dose-dependent manner, reaching statistical significance at doses ≥0.75 mg/rat for LDH and at 1.5 mg/rat for total protein (Figure 2F,G). Among the tested pro-inflammatory cytokines (IL-1β, IL-6, TNF-α, eotaxin, and CINC-3), eotaxin levels were significantly elevated at 3.0 mg/rat and TNF-α levels at doses ≥0.75 mg/rat, both showing clear dose-dependency (Figure 2H,I). However, no significant changes were detected in the other cytokines compared with the control group, with several values below the assay’s detection limit. Histological examination revealed that the pulmonary inflammation was localized to the alveoli, with no evidence of destructive lung damage (Figure 3A–C).

### 3.3. Lung Inflammation at Days 7 and 28 After a Single Intratracheal Instillation of NaF

**Figure 4:**
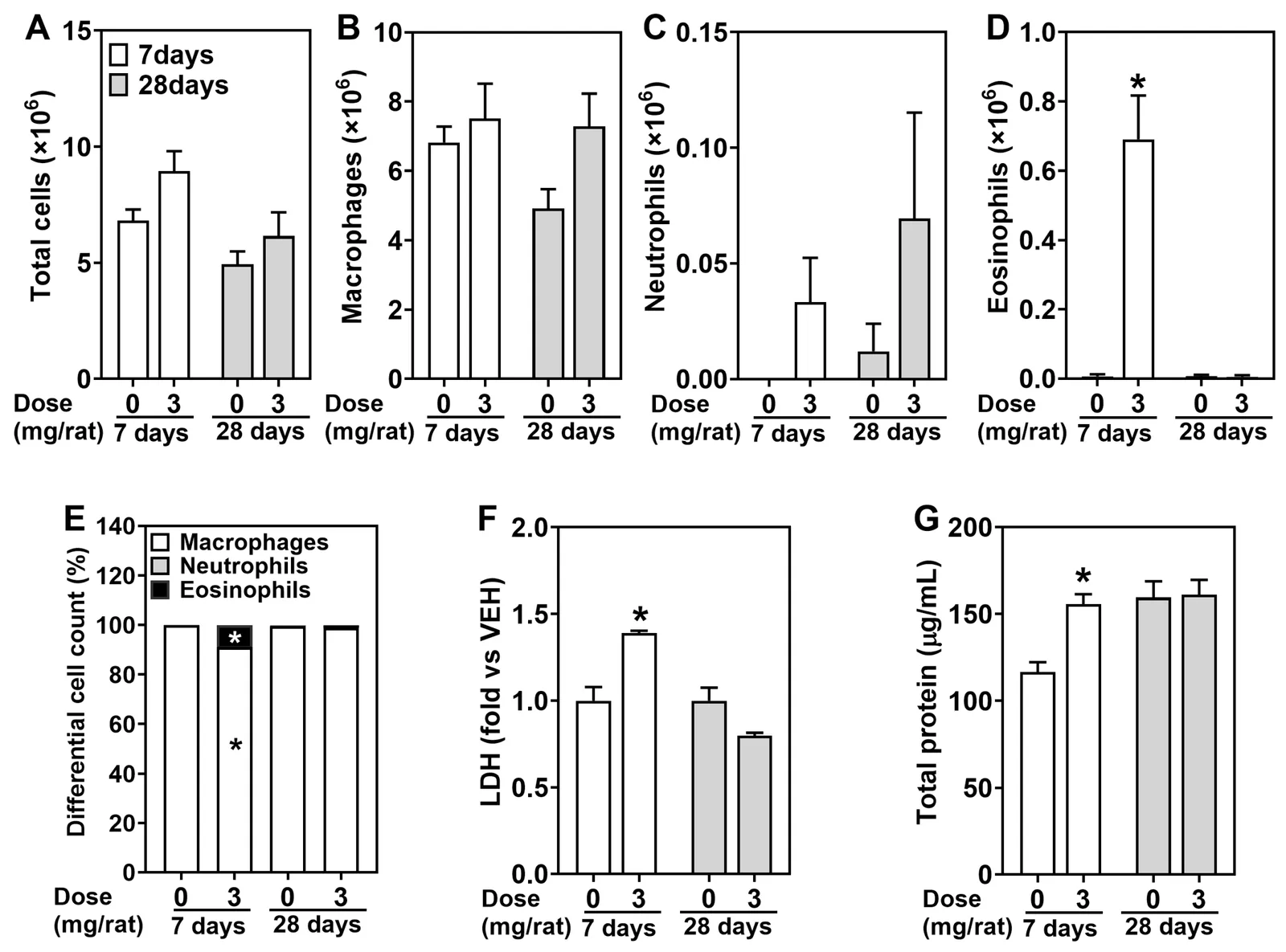
Lung inflammation at 7 and 28 days after a single intratracheal instillation of NaF. Rats were administered NaF at the high dose (3.0 mg/rat), and lung inflammation was assessed by bronchoalveolar lavage fluid (BALF) analysis. (**A**–**E**) Cytological parameters: (**A**) total cell count, (**B**) alveolar macrophage count, (**C**) neutrophil count, (**D**) eosinophil count, and (**E**) percentage of each cell type in BALF. (**F**) Lactate dehydrogenase (LDH) levels. (**G**) Total protein levels. In panels (**A**–**D**) and (**F**,**G**), white and gray bars indicate the 7-day and 28-day groups, respectively. Data are mean ± standard deviation (*n* = 4 per group). * *p* < 0.05 compared to the control group.

Lung inflammation was evaluated at days 7 and 28 after a single intratracheal instillation of NaF (3.0 mg/rat) to determine the reversibility of the inflammatory response. At 7 days, eosinophil counts remained significantly elevated, whereas other cytological parameters showed no significant changes compared with the vehicle control group (Figure 4A–D). The percentage data indicated significant changes only in macrophages (decreased) and eosinophils (increased) at 7 days, with no significant alterations in other cell types (Figure 4E). By 28 days post-instillation, all cytological parameters in BALF had returned to baseline levels, and no significant alterations were noted in any cell type compared with the vehicle control group (Figure 4A–E). LDH and total protein levels also exhibited a mild but significant increase at 7 days, followed by recovery thereafter (Figure 4F,G). The levels of pro-inflammatory cytokines (IL-1β, IL-6, TNF-α, eotaxin, and CINC-3) remained unchanged at both time points compared with the vehicle control group, with several values below the assay’s detection limit. Histopathological examination revealed no evidence of significant pathological damage at either 7 or 28 days after instillation (Figure 3D–F).

### 3.4. Biodistribution of [^18^F]NaF After a Single Intratracheal Instillation in Rats

**Figure 5:**
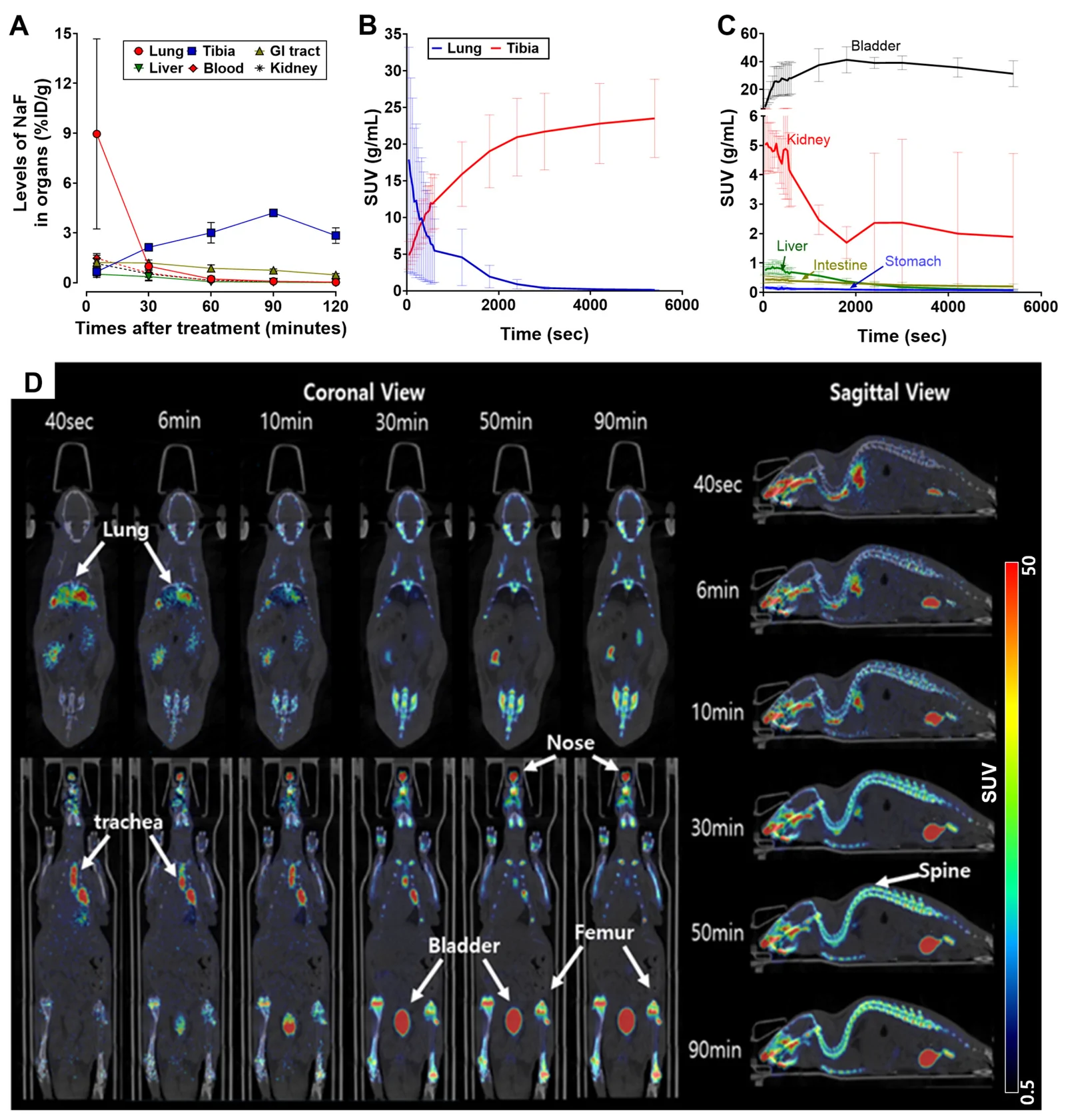
Biodistribution of [^18^F]NaF after a single intratracheal instillation in rats. [^18^F]NaF was intratracheally administered to rats, and radioactivity levels were quantified in various tissues up to 2 h post-instillation. The administered doses were 3.7–7.4 MBq/rat for gamma counting and 29.6–37 MBq/rat for microPET/CT imaging. (**A**) Tissue radioactivity measured by automated gamma counting. The GI tract represents the combined data from the stomach, small intestine, and large intestine. (**B**,**C**) Standardized uptake values (SUVs) of [^18^F]NaF from microPET/CT imaging over 80 min in (**B**) the lungs and bone (tibia), and (**C**) intestine, stomach, liver, kidneys, and bladder. Data are presented as mean ± standard deviation (*n* = 4). (**D**) Representative coronal and sagittal PET/CT images at 40 s, and 6, 10, 30, 50, and 90 min following intratracheal instillation.

Following intratracheal instillation of [^18^F]NaF dispersed in saline, biodistribution analysis using a gamma counter of [^18^F]NaF in rats showed that lung uptake peaked at 5 min (8.95 ± 5.72% ID/g) post-instillation before rapidly declining, indicating fast pulmonary clearance (Figure 5A). In contrast, tibia uptake of [^18^F]NaF increased progressively, reaching a maximum at 90 min (4.20 ± 0.18% ID/g) post-instillation, reflecting systemic redistribution and preferential skeletal deposition. Transient accumulation in the gastrointestinal (GI) tract, liver, kidneys, blood, and other systemic organs remained low, suggesting limited short-term systemic distribution. Dynamic PET/CT SUV analysis of [^18^F]NaF corroborated these findings: lung SUV dropped sharply, whereas tibia SUV of [^18^F]NaF steadily increased, indicating pulmonary clearance and bone accumulation of [^18^F]NaF (Figure 5B). Bladder SUV of [^18^F]NaF rose continuously, consistent with urinary excretion as the primary elimination route (Figure 5C). The kidneys exhibited transient [^18^F]NaF SUV elevation, while the liver, stomach, and intestines maintained consistently low SUV values of [^18^F]NaF (Figure 5C). Representative coronal and sagittal PET/CT images visualized these biodistribution patterns of [^18^F]NaF, showing high early lung radioactivity (40 s) that diminished over time, with increasing accumulation in bone (spine and femur) and bladder (Figure 5D). Collectively, these results demonstrate that [^18^F]NaF undergoes rapid pulmonary clearance, preferential bone accumulation, and renal excretion, with minimal retention in other systemic or gastrointestinal organs.

### 3.5. Biodistribution of [^18^F]NaF After 1 h Nose-Only Inhalation Exposure in Rats

**Figure 6:**
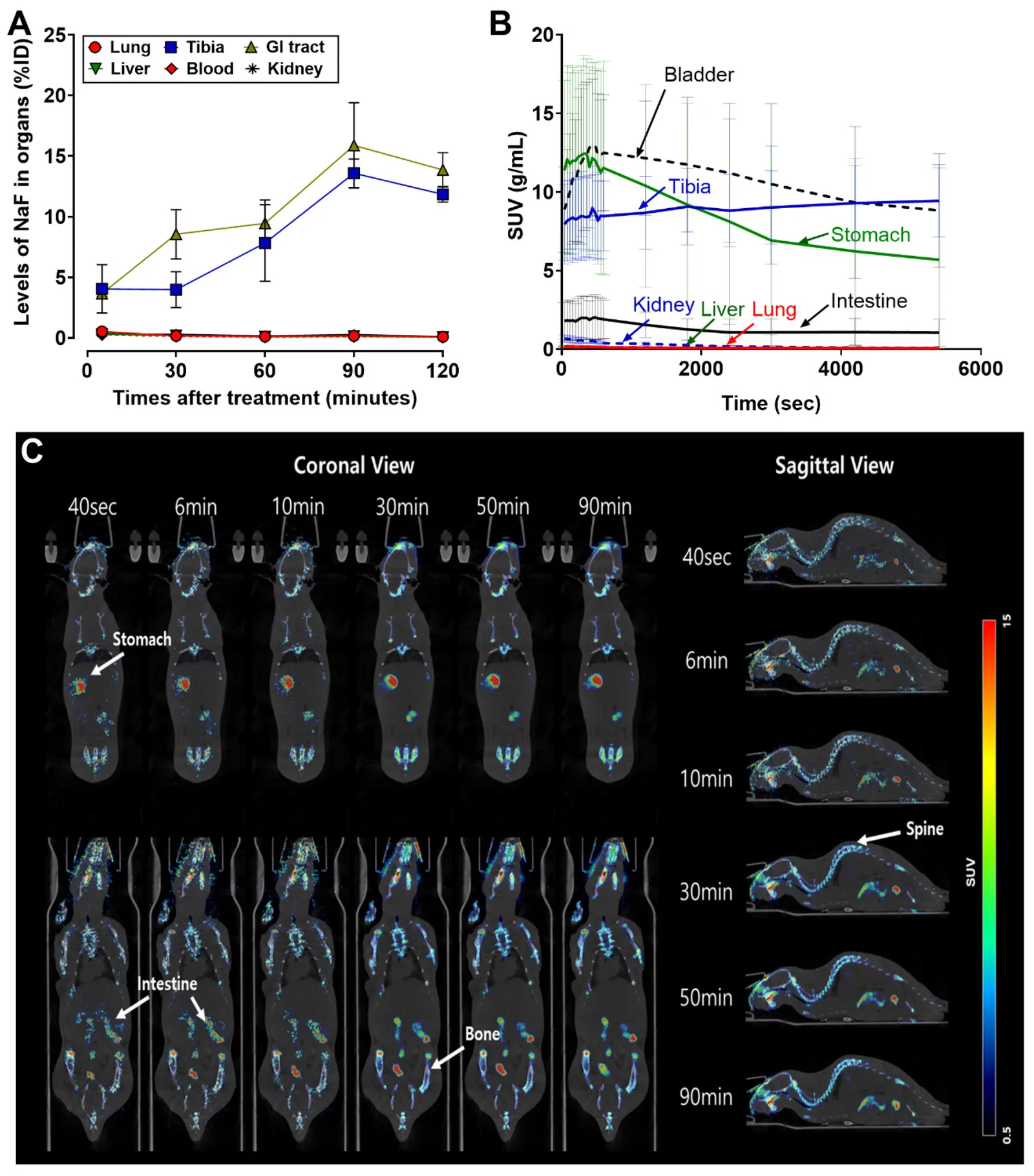
Biodistribution of [^18^F]NaF in rats after 1 h nose-only inhalation exposure. The total amount of [^18^F]NaF detected in rats was 0.74–1.11 MBq/rat for the automated gamma counter and 7.40–11.1 MBq/rat for the microPET/CT imaging. The biodistribution in rats was quantified at various time points for 2 h after 1 h exposure to [^18^F]NaF. (**A**) Radioactivity levels in organs, which were measured by the automated gamma counter. The total activity of the gastrointestinal (GI) tract is the sum of the stomach, small intestine, and large intestine. Data are mean ± standard deviation (*n* = 3). (**B**) Standardized uptake values (SUVs) in organs, measured up to 80 min using microPET/CT images. Data are presented as mean ± standard deviation (*n* = 4). (**C**) PET/CT images of [^18^F]NaF distribution in rats, shown in sagittal and coronal views following 1 h nose-only exposure.

The biodistribution of [^18^F]NaF was analyzed after a 1 h nose-only inhalation exposure using a gamma counter and PET/CT imaging. The mass median aerodynamic diameter (MMAD) and geometric standard deviation (GSD) of the generated aerosols were 0.70 μm and 2.37, respectively. The chamber aerosol concentration was 48.1 MBq/m^3^, and each rat received 0.74–1.11 MBq of the radiotracer. Because a no-carrier-added tracer was used, the chemical mass of NaF in the aerosol was negligible, and the inhalation exposure was therefore quantified by radioactivity rather than chemical mass. In contrast, the toxicity study delivered defined chemical masses of NaF (0.75–3.0 mg/rat) by intratracheal instillation, a bolus route that deposits the majority (>90%) of the administered dose directly in the alveoli, as previously quantified for this technique [19], making it suitable for concentration–response evaluation. Because the tracer-based biodistribution and mass-based toxicity experiments use fundamentally different dose metrics, they are not directly comparable on a single dose scale. Gamma counter analysis of [^18^F]NaF in rats revealed a markedly different uptake pattern compared with the intratracheal instillation study. Specifically, lung levels of [^18^F]NaF remained low and stable throughout the observation, indicating minimal pulmonary retention (Figure 6A). In contrast, tibia and GI tract uptake of [^18^F]NaF progressively increased, reaching the highest levels at 90 min, suggesting systemic absorption and redistribution to bone and gut. Minor uptake of [^18^F]NaF was detected in the liver, kidneys, and blood, without significant temporal variation. Dynamic PET/CT imaging of [^18^F]NaF (Figure 6B,C) confirmed these trends. SUV analysis of [^18^F]NaF showed continuous accumulation in the tibia, stomach, and intestines, while the lung SUV of [^18^F]NaF remained low throughout the imaging period. The bladder showed a steady SUV increase of [^18^F]NaF, consistent with urinary excretion as the primary elimination route. The kidneys exhibited transient uptake, and the liver remained at background levels of [^18^F]NaF (Figure 6B). Representative coronal and sagittal PET/CT images of [^18^F]NaF showed early gastric activity, subsequent intestinal distribution, progressive bone accumulation, and bladder filling over time (Figure 6C). Collectively, these findings demonstrate that inhaled [^18^F]NaF undergoes rapid systemic absorption with preferential deposition in bone and GI tissues, and is primarily cleared via renal excretion, with negligible pulmonary retention.

## 4. Discussion

NaF is a key component of public health measures, including water fluoridation and oral hygiene products, due to its safety and efficacy [20]. Although humans may inhale NaF through exposure to household products, aerosolized fluoridated water, and occupational settings, the toxicological consequences of inhaled fluoride remain insufficiently characterized. Furthermore, the global increase in the use of personal hygiene aerosols to control microbial contamination underscores the need for a more precise and comprehensive evaluation of their safety in humans and the environment [4]. In this study, we investigated the pulmonary toxicity of NaF to identify potential toxic effects and their related mechanisms. In addition, we evaluated the biodistribution of [^18^F]NaF using intratracheal instillation and nose-only inhalation models to better understand NaF behavior under two distinct pulmonary exposure scenarios. Importantly, the PET study used tracer-level [^18^F]NaF for biodistribution analysis, whereas the intratracheal instillation study used mg-level NaF to evaluate toxicological responses. These two approaches were therefore designed to be complementary, with instillation used for hazard identification and inhalation for realistic biodistribution, rather than to define a continuous dose-response relationship; together they provide an integrated view of the pulmonary hazard and systemic fate of inhaled NaF that neither could provide alone.

Several methodological differences between the toxicity and biodistribution experiments warrant clarification, as they were deliberate design choices rather than inconsistencies. First, with respect to the exposure route, the biodistribution of [^18^F]NaF was characterized by both intratracheal instillation and nose-only inhalation (Figure 5 and Figure 6). The instillation route thus matches the delivery method used in the toxicity study, providing a route-consistent link between the two experiments, while the inhalation route additionally captures the biodistribution under a realistic exposure scenario. The toxicity and biodistribution datasets are therefore not built on mismatched routes. Second, with respect to the dose range, the difference in dose metric is intrinsic to the methods and does not permit, nor did we attempt, a direct numerical comparison. The toxicity study used defined chemical masses of NaF (0.75–3.0 mg/rat) because concentration–response hazard identification requires known chemical doses, whereas the biodistribution study used a no-carrier-added [^18^F]NaF radiotracer, for which radioactivity (MBq), not chemical mass, is the appropriate dose metric; the negligible chemical mass of the tracer is precisely what allows it to report physiological distribution without perturbing it. Accordingly, the two datasets were interpreted independently on their own dose scales and were never equated. Third, with respect to the sampling time points, the two experiments were designed to resolve processes on different time scales. The toxicity study sampled at days 1, 7, and 28 to track the onset, peak, and resolution of the pulmonary inflammatory response, whereas the biodistribution study sampled over minutes to hours to resolve the rapid absorption, systemic redistribution, and clearance of fluoride. Each sampling schedule was matched to the kinetics of the process being measured, and the delayed inflammatory time course is in fact consistent with, and explained by, the rapid clearance documented in the biodistribution study. Taken together, these design choices make the two experiments complementary and mutually reinforcing rather than contradictory.

Our results demonstrated that NaF induces transient but significant pulmonary inflammation when delivered directly into the lungs. At 24 h post-intratracheal instillation, total BALF cell counts increased in a dose-dependent manner, with marked elevations in neutrophils and eosinophils, alongside increases in LDH and total protein levels, indicating acute cytotoxic injury and increased alveolar–capillary permeability [21,22]. Although the BALF analysis revealed a pronounced inflammatory response, the accompanying histopathological changes were only mild, without destructive tissue damage. This apparent discrepancy reflects the well-recognized difference in sensitivity between the two endpoints rather than a true inconsistency, because BALF analysis is considerably more sensitive than conventional hematoxylin and eosin histopathology for detecting acute pulmonary inflammation. It is also consistent with the rapid pulmonary absorption and clearance of NaF, which limit sustained structural injury after a single exposure. For this reason, the objective and quantitative assessment of pulmonary inflammation is provided primarily by the BALF endpoints; this rationale underlies the 2018 revision of the OECD inhalation toxicity test guidelines (TG 412 and TG 413), which made BALF analysis a mandatory endpoint to complement the more subjective histopathological evaluation [23,24]. Accordingly, semi-quantitative scoring of the near-normal lung tissue would provide little additional discriminatory information beyond the quantitative BALF data.

The eosinophilia observed in BALF may be associated with multiple mechanisms in the lung. One possible explanation is the passive release of intracellular eotaxin from damaged cells, a phenomenon also reported with several nanomaterials, including zinc oxide, copper oxide, and nickel oxide [21,25]. In addition, active secretion by intact epithelial cells in response to inflammatory mediators such as TNF-α may also have contributed to the elevated eotaxin detected in BALF [26]. Previous studies on NaF respiratory toxicity are scarce; however, one report reported neutrophilia without eosinophilia in rats given a single intratracheal instillation of 400 μg/rat NaF, which may have been due to the lower dose used compared with that in the present study [27]. Thus, our findings demonstrate that a single high-dose intratracheal instillation of NaF can induce acute eosinophilic inflammation, potentially exacerbating eosinophil-associated respiratory diseases, highlighting its potential pulmonary hazard. The upstream mechanisms linking NaF exposure to the observed release of TNF-α and eotaxin were not directly investigated in this study, but they can be inferred from the known cellular toxicology of fluoride. In pulmonary cells, NaF increases NADPH oxidase 4 (NOX4)-derived reactive oxygen species, and this oxidative stress activates a p53/death receptor 5 (DR5) axis that drives caspase-mediated apoptosis [28]; fluoride also causes mitochondrial dysfunction that further amplifies reactive oxygen species generation and caspase-dependent cell death [29]. Such oxidative and cytotoxic injury can, in turn, activate downstream inflammatory signaling and promote the release of pre-formed mediators such as eotaxin from damaged cells, consistent with the acute inflammatory response observed here. Nonetheless, the specific upstream pathways (e.g., NOX4/ROS, p53/DR5, and mitochondrial apoptotic cascades) driving NaF-induced pulmonary inflammation were not measured in the present study and warrant dedicated mechanistic investigation in future work.

Although asthma is often driven by antigen-specific allergic sensitization, a non-immunologic form of the disease, termed irritant-induced asthma, can develop when inhaled irritants directly injure the airway epithelium without prior immunological sensitization [30]. The acute, cytotoxicity-driven eosinophilic response observed here is more consistent with such a non-allergic, epithelial injury-driven mechanism, and aerosolized NaF might therefore contribute to eosinophil-associated airway inflammation through direct epithelial cytotoxicity rather than through classical allergic sensitization. Whether repeated exposure sustains this eosinophilic airway inflammation or progresses to chronic pathological changes remains to be determined in repeated-exposure studies. However, intratracheal instillation delivers a bolus dose directly to the lungs, bypassing upper-airway filtration and resulting in substantially higher initial pulmonary deposition than that of realistic inhalation exposure. Therefore, the lung inflammation data in this study should be interpreted primarily as evidence of pulmonary hazard potential rather than as direct risk information for realistic inhalation exposure.

The rapid pulmonary clearance of NaF is not inconsistent with the delayed and sustained inflammatory response. During the brief period when fluoride is absorbed across the pulmonary epithelium, a transient intracellular spike in fluoride concentration is sufficient to trigger cytotoxic injury and induce inflammatory mediators such as TNF-α and eotaxin. Once initiated, this response propagates through a self-amplifying cytokine cascade (for example, TNF-α-driven eotaxin release and eosinophil recruitment) that persists after the triggering agent has been cleared, accounting for the peak of inflammation at day 1 and the elevated eosinophil counts up to day 7. Importantly, this sustained inflammation represents a reversible host response and a process of resolution and repair rather than progressive structural injury, as it was not accompanied by destructive histopathological changes and had fully resolved by day 28. Although repeated pulmonary exposure studies with NaF were not conducted here, repeated oral NaF administration in rats has been shown to cause progressive and irreversible lung alterations, including alveolar septal thickening, fibrosis (collagen deposition), structural remodeling, epithelial hyperplasia, and alveolar damage [31,32]. These findings indicate that the lung may be a target organ of systemic fluorosis and raise the possibility that repeated or chronic inhalation exposure could contribute to similar changes, although this remains a hypothesis that requires confirmation in dedicated inhalation repeated-exposure studies. However, our data also indicate that short-term NaF inhalation is more likely to cause a reversible and tolerable inflammatory response, as all inflammation-related parameters returned to baseline by 28 days post-exposure.

Beyond pulmonary toxicity, we found that [^18^F]NaF is rapidly absorbed from the lungs and distributed systemically, with preferential skeletal deposition. Following intratracheal instillation, lung uptake of [^18^F]NaF peaked at 5 min and then rapidly declined, accompanied by progressive accumulation in bone. In contrast, the nose-only inhalation data of [^18^F]NaF were obtained only after completion of the 1-h exposure period and therefore reflect post-exposure distribution rather than the earliest phase of pulmonary absorption and systemic transfer. Fluoride’s high skeletal affinity is primarily due to ionic substitution of hydroxyl groups in hydroxyapatite to form fluorapatite, which is more stable and acid-resistant [33]. Furthermore, fluoride promotes osteoblastic activity via mitogen-activated protein kinase (MAPK) and Wnt/β-catenin signaling pathways, and suppresses osteoclastic bone resorption by modulating the receptor activator of nuclear factor of kappa-light-chain-enhancer of activated B cells ligand/osteoprotegerin pathway [34]. Importantly, [^18^F]NaF is a clinically established bone PET tracer in humans, and its documented human biodistribution, characterized by rapid clearance from blood, high first-pass skeletal uptake governed by regional blood flow and bone surface area, and predominant renal and urinary excretion, closely parallels the biodistribution observed here in rats [35]. This concordance supports the physiological relevance of our biodistribution findings to humans and indicates that the lung may serve as an efficient route of systemic fluoride absorption in humans as well. These biodistribution findings are unlikely to be confounded by the isoflurane anesthesia used during the intratracheal procedures. Although isoflurane can be defluorinated to inorganic fluoride, its metabolism is minimal (~0.2%) and increases plasma inorganic fluoride only marginally (on the order of 1 µM) [36]. Furthermore, because [^18^F]NaF was administered as a no-carrier-added tracer, the skeletal fluoride-binding capacity vastly exceeds the amounts of both the tracer and any isoflurane-derived fluoride, so competition for bone binding sites does not occur, and skeletal tracer uptake is governed by regional blood flow and bone surface area rather than by saturation [35].

This study has several limitations. (1) The intratracheal instillation and nose-only inhalation paradigms had an asymmetric temporal design. Given the rapid absorption and systemic clearance of [^18^F]NaF, a substantial and unquantified fraction of the inhaled tracer may already have been absorbed, distributed, and partially excreted before the first measurement was taken. Therefore, the inhalation data primarily represent the post-exposure distribution phase and do not capture the full kinetic profile. (2) The inhalation gamma-counter biodistribution analysis was based on a small final sample size (*n* = 3 per time point), which may have limited sensitivity to detect subtle differences. (3) The aerosol size used in the inhalation study (MMAD: 0.70 µm) and hydrophilicity may have influenced the deposition pattern, with a fraction of inhaled [^18^F]NaF depositing in the extrathoracic airways rather than the deep lung [37,38]. Material deposited in the nasopharyngeal region could subsequently undergo mucociliary clearance and be swallowed, thereby contributing to the gastrointestinal uptake observed after nose-only inhalation [39]. (4) Pulmonary toxicity was assessed only by intratracheal instillation and not by inhalation. Intratracheal instillation was used because it permits precise control of the delivered pulmonary dose for concentration-response assessment. Evaluation of NaF toxicity under realistic inhalation exposure was beyond the scope of the present study and warrants further investigation in dedicated inhalation toxicity studies. Nevertheless, the two exposure paradigms provide complementary information on [^18^F]NaF biodistribution under different pulmonary exposure conditions. (5) Only male rats were used in this study because estrogen can modulate pulmonary inflammatory responses [40] and because occupational fluoride exposure has historically predominated in male-dominated settings; consequently, potential sex-specific differences in the pulmonary toxicity and biodistribution of NaF were not evaluated and warrant investigation in future studies including both sexes.

Although fluoride has dental benefits, excessive accumulation in bone, particularly under chronic or repeated exposure, raises concerns about its toxicological effects [41]. PET/CT imaging confirmed time-dependent bone uptake and prominent bladder activity, indicating renal excretion as the primary route of clearance. Additional uptake of [^18^F]NaF in the GI tract, liver, and kidneys supports mixed excretion pathways, whereas accumulation in soft tissues, such as the heart and brain, was minimal. The high efficiency with which fluoride transits from the pulmonary surface into systemic circulation highlights the lung as a potentially important route of fluoride absorption. This is particularly concerning for aerosolized consumer products, which can lead to repeated low-level exposures in poorly ventilated environments [42]. These findings have important implications for chemical safety evaluation. Current regulatory assessments of fluoride focus largely on ingestion, potentially underestimating inhalation risks. Our results indicate that NaF is efficiently absorbed via the lungs and distributed systemically; therefore, repeated or long-term exposure, even at low doses, may increase the risk of systemic fluoride toxicity, including skeletal accumulation and possible reproductive or neurological effects reported in oral exposure studies [43,44]. This concern is heightened in occupational settings or among individuals who frequently use aerosolized fluoride-containing products [45]. Although this biodistribution study provided valuable information on the systemic fate of inhaled fluoride, the tracer dose used differed from the chemical doses used in the toxicity study, and the effect of dose on the pulmonary and systemic response therefore warrants further study.

The NaF dose used in this study (3 mg/rat, approximately 15 mg/kg) is lower than the total daily occupational exposure limits set by the US OSHA and NIOSH guidelines (i.e., 2.5 mg/m^3^, 8-h time-weighted average), equivalent to roughly 20 mg/day in humans [14,15]. However, this occupational limit corresponds to ~0.33 mg/kg/day in a 60 kg human, meaning the experimental dose here exceeds typical daily occupational exposure by more than an order of magnitude. Nonetheless, in certain high-risk occupational environments, such as aluminum smelters or phosphate fertilizer plants, airborne fluoride concentrations can significantly exceed OSHA limits, with reported inhalation exposures reaching hundreds of milligrams per day [16]. Thus, the 3 mg/rat dose used here may approximate extreme occupational exposure scenarios. Moreover, the estimated daily fluoride intake from drinking water and toothpaste for the general population (approximately 1–3 mg/day) based on the maximum allowable concentration (4 mg/L) and recommended fluoridation level (0.7 mg/L) of the Environmental Protection Agency (EPA) [46] is comparable to the absolute dose used in our experiment. Because ingested fluoride is systemically absorbed and can accumulate in various organs, including the lungs [16], combined oral and inhalation exposure could produce lung fluoride levels similar to those observed at the 3 mg/rat dose in this study. Therefore, future human health risk assessments should incorporate realistic combined-exposure scenarios to estimate fluoride accumulation and the associated health risks in target organs.

## 5. Conclusions

Inhalation exposure to NaF from consumer products and certain occupational environments is a realistic scenario, yet current toxicity and biodistribution data remain insufficient to assess the associated health risks. In this study, intratracheal instillation of NaF induced significant but reversible neutrophilic and eosinophilic inflammation, likely associated with cytotoxicity-driven release of TNF-α and eotaxin, suggesting that repeated or high-concentration exposure may contribute to eosinophil-associated airway inflammation. PET/CT imaging and biodistribution study of [^18^F]NaF demonstrated rapid pulmonary clearance and preferential skeletal deposition of [^18^F]NaF following intratracheal instillation, whereas the nose-only inhalation study of [^18^F]NaF showed post-exposure systemic distribution with low pulmonary retention and accumulation in bone and excretory organs. Because inhalation measurements were initiated only after the exposure period, direct kinetic comparisons between the two exposure routes should be interpreted with caution. Although the experimental dose was higher than typical daily human exposures, combined inhalation and oral routes may contribute to systemic fluoride burden, with potential implications for pulmonary and systemic toxicity. Therefore, future human health risk assessments should incorporate realistic combined exposure scenarios to more accurately estimate fluoride accumulation and associated toxicities in target organs.

## Data Availability

All data generated or analyzed during this study are included in this published article. Additional datasets are available from the corresponding author upon reasonable request.

## Abbreviations

The following abbreviations are used in this manuscript:

NaF	Sodium Fluoride
BALF	Bronchoalveolar lavage fluid
PET/CT	Positron emission tomography/computed tomography
[^18^F]NaF	Fluorine-18 radiolabeled sodium fluoride
COVID-19	Ccoronavirus disease 2019
QMA	Quaternary methyl ammonium
radio-TLC	Radio-thin-layer chromatography
KIRAMS	Korea institute of radiological and medical sciences
IACUC	Institutional animal care and use committee
SD	Sprague-Dawley
DW	Distilled water
PBS	Phosphate-buffered saline
LDH	Lactate dehydrogenase
BCA	Bicinchoninic acid
IL	Interleukin
TNF	Tumor necrosis factor
CINC-3	Cytokine-induced neutrophil chemoattractant-3
ELISA	Enzyme-linked immunosorbent assay
3D-OSEM	3D ordered subset expectation maximization
PMOD	PET model organism database
SUV	Standardized uptake value
VOI	Volume of interest
ANOVA	Analysis of variance
GI	Gastrointestinal
MMAD	Mass median aerodynamic diameter
GSD	Geometric standard deviation
OSHA	Occupational safety and health administration
NIOSH	National institute for occupational safety and health
EPA	Environmental protection Agency
